# Valorization of Bio-Oil Aqueous Fractions Through Oxidative Steam Reforming over Co/CeO_2_-SBA-15 Catalysts: From Single Model Compounds to Complex Mixtures

**DOI:** 10.3390/nano16020085

**Published:** 2026-01-08

**Authors:** Carlos A. Chirinos, Arturo J. Vizcaíno, José A. Calles, Alicia Carrero, Pedro J. Megía

**Affiliations:** 1 Chemical and Environmental Engineering Group, Universidad Rey Juan Carlos, c/Tulipán s/n, 28933 Móstoles, Spain; carlos.chirinos@urjc.es (C.A.C.); arturo.vizcaino@urjc.es (A.J.V.); alicia.carrero@urjc.es (A.C.); 2 Instituto de Investigación de Tecnologías para la Sostenibilidad, Universidad Rey Juan Carlos, c/Tulipán s/n, 28933 Móstoles, Spain

**Keywords:** hydrogen production, biomass, bio-oil aqueous phase, model compound mixtures

## Abstract

This study investigates the oxidative steam reforming (OSR) of simulated bio-oil aqueous fractions using Co/CeO_2_-SBA-15 catalysts. Five representative compounds—methanol, acetic acid, hydroxyacetone, phenol, and furfural—were evaluated to assess their reactivity for hydrogen production. Aliphatic compounds achieved nearly complete conversion and stable hydrogen yields, while aromatic structures led to lower conversion and higher coke formation. Furfural exhibited higher reactivity than phenol due to its furan ring and aldehyde group. Catalysts with 10 and 20 wt.% Ce showed similar activity, but Co/20CeO_2_-SBA-15 presented lower hydrogen yield. For this reason, next experiments of OSR of model compound mixtures were carried out only with Co/10CeO_2_-SBA-15. To approach real bio-oil complexity, ternary and quinary mixtures were tested. High conversion and hydrogen yield were maintained over 50 h when the ternary mixture (methanol, hydroxyacetone, and acetic acid) was fed. When the quinary mixture was used as feedstock, which includes furfural and phenol, lower conversions were obtained for these compounds compared to aliphatic ones, although conversions remained above 80% after 50 h (88.9% for furfural and 82.6% for phenol). These results highlight Co/10CeO_2_-SBA-15 as a viable catalyst for bio-oil aqueous fraction valorization under OSR conditions.

## 1. Introduction

The growing global energy demand has remained strongly dependent on fossil fuels, accounting for over 80% of primary energy worldwide. In 2023, oil was the dominant source, representing 32% of global consumption, followed by coal at 26% and natural gas at 23% [1]. This dependence has significant environmental, economic and geopolitical impacts. The non-renewable characteristics of fossil fuels, along with their progressive geographic concentration and various international conflicts, have influenced the different fluctuations in the energy markets [2]. Furthermore, fossil fuels contribute significantly to global pollution, primarily through the emission of greenhouse gases, especially CO_2_. These emissions have severe environmental consequences, driving global warming at the same time that affects weather patterns, ecosystems, and human health [3]. To face this challenge, in recent decades, the development of renewable energy sources and the advancement of more sustainable production technologies have increased. This effort aims to diversify the energy mix while reducing the carbon footprint. In this context, biorefining emerges as one of the most important strategies [4]. Based on the oil refinery model, a biorefinery is defined as a sustainable bioprocess in which biomass resources—such as agricultural, forestry, or urban residues—are efficiently used as renewable feedstocks for the production of biofuels, chemical intermediates, and high-value-added materials. The main objective is to maximize biomass valorization via multistage processing schemes that integrate thermochemical, biotechnological, and catalytic pathways to obtain valuable products [5,6]. Among the available technologies integrated in a biorefinery, biomass fast pyrolysis has been recognized as an efficient method for the thermochemical transformation of lignocellulosic feedstocks into biofuel products [4,7]. This process is carried out under oxygen-free conditions at temperatures ranging from 400 to 600 °C. It yields three main fractions: non-condensable gases (CO, CO_2_, H_2_, CH_4_); biochar, a carbon-rich solid phase; and a highly oxygenated liquid known as bio-oil [7]. Bio-oil, which can represent up to 70% of the total product distribution, is a complex mixture of heterogeneous organic compounds, including alcohols, carboxylic acids, aldehydes, ketones, furans, phenolic compounds, anhydrosugars, esters, and long-chain hydrocarbons derived from lignin depolymerization [8,9]. The final composition strongly depends on both the feedstock characteristics and the operating conditions used during pyrolysis [10].

Bio-oil is considered a potential alternative to conventional liquid fuels; however, it exhibits unfavorable properties, including high viscosity, thermal and chemical instability, high acidity, and low heating value [11]. As a result, its direct application is significantly limited. Bio-oil can be segregated into two phases by adding water: an organic fraction, which can be upgraded for the production of advanced biofuels or used directly as a low-quality fuel, and an aqueous fraction with low value. Although the aqueous phase contains light oxygenates, it is mainly composed of water, thus limiting its potential use [9]. Nonetheless, from a circular economy perspective, the aqueous fraction is considered an attractive resource because its catalytic transformation can yield high-value-added products, including hydrogen via reforming processes [12]. Due to the complex mixture of oxygenated hydrocarbons present in the aqueous phase, many researchers have explored its valorization through steam reforming of model compounds [13,14,15,16]. These investigations have demonstrated that the reactivity of individual compounds, the transformation pathways, the product distribution and the tendency to coke formation are determined by the chemical structure of the model compound used, the operational conditions, and the catalyst used. Although the use of model compounds offers valuable insights, it cannot reproduce the inherent complexity of real bio-oil aqueous fraction, where different oxygenated compounds coexist and may interact, potentially affecting catalytic performance, product distribution and carbon deposition. Thus, incorporating a broader range of representative compounds from each major family present in bio-oil aqueous fractions—such as alcohols, ketones, acids, aldehydes, and phenolics—allows for a more accurate simulation of key phenomena in reforming processes, including competitive adsorption, reaction pathways, and potential interaction effects. This facilitates a more comprehensive understanding of catalyst behavior under conditions that closely resemble those of real bio-oil aqueous fractions. Depending on the biomass feedstock used for the production of bio-oil, different compositions can be obtained in the aqueous fraction. Bergem et al. [17] identified alcohols, carboxylic acids, and ketones as the predominant functional groups in the bio-oil aqueous fraction derived from fast pyrolysis of red oak at 500 °C. Among these, methanol, acetic acid and hydroxyacetone were reported as the major individual compounds within each respective family, making them suitable candidates for formulating a ternary mixture that closely resembles the composition of this fraction. Based on the composition reported by Fermoso et al. [18], obtained during the catalytic fast pyrolysis of partially de-ashed wheat straw, carboxylic acids, ketones/ethers, furans and oxygenated aromatics were identified as the dominant chemical families in the bio-oil aqueous fraction. To better simulate the complexity of this fraction, phenol and furfural can be incorporated into the previously proposed ternary mixture, as representative compounds of oxygenated aromatics and furans, respectively. Despite the advantages of combining different model compounds for catalyst testing in reforming processes, only a limited number of studies have investigated mixtures of model compounds [16,19,20], and even fewer have examined the catalytic behavior using real fractions [21].

Given that conventional steam reforming is highly energy-intensive, oxidative steam reforming (OSR) arises as a suitable technology for the valorization of these compounds [21,22]. The OSR involves a combination of an endothermic steam reforming reaction along with exothermic oxidative reactions, leading to a significant reduction in the energy requirements apart from limiting the coke formation on the catalytic surface [23]. The overall reaction scheme is shown in Equation (1):

(1) $$C_{n}H_{m}O_{k}+\alpha O_{2}+\left(2n-2\alpha -k\right)\;H_{2}O\to \;(2n-2\alpha -k\;+m/2)\;H_{2}+n{\mathrm{CO}}_{2}$$

Additionally, the development of stable, active and hydrogen-selective catalysts is essential for optimizing hydrogen production during the oxidative steam reforming of bio-oil aqueous fractions. However, one of the main challenges remains in the catalytic deactivation along reforming reactions [24]. This deactivation can result from carbon deposition, active phase sintering, and, specifically in oxidative steam reforming, particularly the active phase re-oxidation [25].

To maximize catalytic performance and avoid deactivation mechanisms, considerable research has been conducted on the development of supports with structurally controlled properties. Mesoporous materials such as MCM-41 [26], KIT-6 [27], or SBA-15 [28,29] have been extensively investigated for their capacity to enhance the dispersion of the active phase, apart from allowing the transport of reactants and products within the support structure. Among them, SBA-15 stands out due to its balanced combination of high surface area, uniform pore distribution, and good thermal stability [29]. These features allow a well-controlled impregnation of the active phase, promoting the formation of active metal sites, while minimizing carbonaceous deposits by reducing diffusional limitations.

However, the support properties alone may not be sufficient to ensure the optimal catalytic performance. In this regard, numerous studies have demonstrated that incorporating metal oxides as support modifiers can significantly improve catalyst functionality [30]. Metal oxides such as MgO [31], ZrO_2_ [32], La_2_O_3_ [33], CeO_2_ [34], or Al_2_O_3_ [35] have been incorporated into the SBA-15 support to modify the acidity, stabilize the metal phase, or induce new metal-support interactions. Among them, CeO_2,_ apart from being active in the water–gas shift reaction, has been extensively studied for its ability to generate oxygen vacancies, which promote reactions of intermediate partial oxidation [32,34,36,37,38]. In previous research [38], our group analyzed in detail the effect of Ce incorporation at different percentages (0, 5, 10, 20, and 30 wt.%) on Co/SBA-15 catalysts in the acetic acid oxidative steam reforming (AAOSR). The results showed that catalysts containing 10 and 20 wt.% of Ce as support modifier achieved a suitable balance of acetic acid conversion, H_2_ yield, catalytic stability, and resistance to coke formation. Beyond the properties of support, the selection of an appropriate active phase is a critical factor in determining catalytic behavior. Among the most extensively investigated active phases, transition metals—such as cobalt—being cheaper than noble metals, have shown promising performance due to their capacity to facilitate the C–C, C–H and C–O bond cleavage, which is an essential requirement in reforming reactions [39,40,41]. Compared to other non-noble metals, cobalt exhibits higher resistance to coke formation and operates stably across a wide range of temperatures [42]. Nevertheless, its catalytic performance is highly dependent on the active phase dispersion over the support and the metal-support interactions involved [43].

Based on the above, the present work aims to advance the understanding of Co/CeO_2_-SBA-15 catalysts by evaluating formulations with 10 and 20 wt.% Ce in the oxidative steam reforming of representative model compounds and their mixtures as an approach to the complex composition of bio-oil aqueous fraction. For this purpose, five representative oxygenated compounds—methanol, acetic acid, hydroxyacetone, phenol, and furfural—were selected. Additionally, ternary and quinary mixtures were also evaluated to reflect real feed conditions better. Unlike previous studies focused on single compounds, this approach provides a broader view of how compounds with different chemical functionalities interact under reforming conditions, offering new insights into catalyst behavior with complex feeds.

## 2. Materials and Methods

### 2.1. Catalyst Preparation and Characterization

The Co/CeO_2_-SBA-15 catalysts used in this study were synthesized and characterized following the procedure described in our previous work [38] and are therefore only briefly summarized here. SBA-15 (ACS Material, Pasadena, CA, USA) was employed as support due to its high surface area and ordered mesoporous structure. Cerium was incorporated via incipient wetness impregnation using aqueous Ce(NO_3_)_3_·6H_2_O (Sigma-Aldrich, St. Louis, MO, USA) solutions, to obtain loadings of 10 and 20 wt.%, followed by calcination at 550 °C. Cobalt was subsequently incorporated via a second impregnation step using Co(NO_3_)_2_·6H_2_O (Sigma-Aldrich, St. Louis, MO, USA) to obtain a final Co loading of 7 wt.%. The resulting materials were denoted as Co/xCeO_2_-SBA-15, where x represents the theoretical Ce content.

Catalyst characterization was performed using different physicochemical techniques. Elemental composition was determined by ICP-OES using an Agilent 5800 VDV spectrometer (Agilent, Santa Clara, CA, USA), after acid digestion. Textural properties were assessed via N_2_ adsorption–desorption isotherms at 77 K using a Micromeritics TRISTAR 3000 analyzer (Micromeritics, Norcross, GA, USA), with specific surface area and pore size distribution calculated using BET and BJH methods, respectively. XRD patterns recorded on a Philips X’PERT PRO diffractometer (Philips, Eindhoven, The Netherlands) allowed the estimation of the mean cobalt crystallite using the Scherrer equation, considering the full width at half maximum (FWHM) of the most intense diffraction peak. X-ray photoelectron spectroscopy (XPS) measurements were performed using a PHI VersaProbe II Scanning Microprobe (Physical Electronics, Chanhassen, MN, USA) at the Central Research Facilities (SCAI), University of Málaga. The analysis employed a monochromatic Al Kα source (1486.6 eV) operating at 15 kV and 25 W, with a beam diameter of approximately 200 µm. Coke deposition on the spent catalysts was evaluated by thermogravimetric analysis (TGA). The measurements were carried out using a Mettler Toledo TGA-DSC instrument (Mettler Toledo, Greifensee, Switzerland) under an air flow.

### 2.2. Catalytic Test

Five oxygenated compounds were selected to be representative of the main compound families generally identified in the bio-oil aqueous phase: acetic acid (carboxylic acid), methanol (alcohol), hydroxyacetone (ketone), phenol (oxygenated aromatic), and furfural (furans and aldehydes). These compounds were studied individually and as part of two representative mixtures—a ternary and a quinary mixture—to better simulate real conditions. In the oxidative steam reforming of model compounds, the steam-to-carbon ratio (S/C) was established as double of the required stoichiometric ratio in the steam reforming reaction with acetic acid (2.00), methanol (2.00), and hydroxyacetone (2.67). However, for phenol and furfural, it was necessary to increase the S/C ratios to 11 and 13.2, respectively, to overcome their lower water solubility.

Catalytic tests were conducted in a continuous-flow fixed-bed reactor (Microactivity-Pro, PID Eng&Tech., Alcobendas, Madrid, Spain) operating at 550 °C and atmospheric pressure. The reactor, a stainless-steel tubular unit (ID: 9.2 mm, length: 300 mm), was loaded with 300 mg of catalyst, which was reduced in situ under pure H_2_ (30 mL/min) at 600 °C for 30 min. After activation, the system was stabilized under N_2_ until the target temperature was reached. Feed solutions—either individual model compounds or representative mixtures—were fed via an HPLC pump at 0.075 mL/min. The O_2_ supply was adjusted to maintain an O_2_/C molar ratio of 0.0375 and a total gas flow of 60 mL/min. Reaction products were separated into condensable and non-condensable fractions. Condensates were collected at 4 °C and analyzed by GC-FID (Agilent 7820A, CP-WAX 52 CB column; Agilent, Santa Clara, CA, USA). At the same time, permanent gases were monitored online using a MicroGC 490 (Agilent, Santa Clara, CA, USA) equipped with Molecular Sieve 5A and PoraPlot U columns (Agilent, Santa Clara, CA, USA). Catalytic performance was assessed through conversion, hydrogen yield, and product selectivity, enabling a comparative evaluation of individual compounds and complex mixtures under OSR conditions.

The catalytic performance was evaluated by determining the conversion of the model compound (X), the hydrogen yield (YH_2_), and the selectivity to carbon-containing products (S_CO_, SCH_4_, SCO_2_, SC_3_H_6_O) calculated according to the following expressions:

(2) $$X_{\mathrm{reactant}\;}(\%)=\frac{F_{\mathrm{reactant},\mathrm{in}}\;-\;F_{\mathrm{reactant},\mathrm{out}}}{F_{\mathrm{reactant},\mathrm{in}}}\cdot 100$$

(3) $$Y_{H_{2}}\;(\%)=\frac{F_{H_{2},\mathrm{out}}}{n_{i}.F_{\mathrm{reactant}}}\cdot 100$$

(4) $$S_{i,\mathrm{carbon}\text{-}\mathrm{containing}\;\mathrm{products}}\;(\%)=\frac{F_{i,\mathrm{carbon}\text{-}\mathrm{containing}\;\mathrm{products}}}{a_{i}.(F_{i,\mathrm{in}}\;-F_{i,\mathrm{out}})}\cdot 100$$

where F represents the molar flow rate of the i species at either the inlet (in) or the outlet (out) of the reactor. The term n_i_ corresponds to the stoichiometric coefficient for the maximum hydrogen generation from each reactant, while a_i_ denotes the carbon-based stoichiometric factor used to relate each product to the feed.

## 3. Results and Discussion

### 3.1. Catalyst Characteristics

Detailed discussion about the characterization of Co/10CeO_2_-SBA-15 and Co/20CeO_2_-SBA-15 catalysts is reported elsewhere [38]. Table 1 summarizes the main physicochemical properties for these catalysts, such as elemental composition, textural properties, reducibility of the Co phase, and the Co^0^ mean crystallite size.

**Figure 1:**
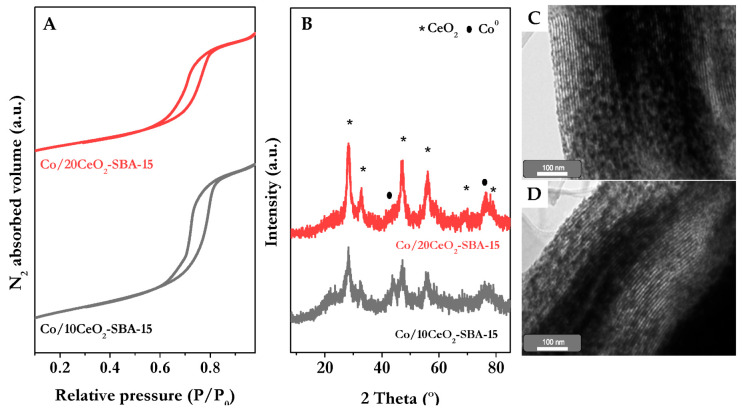
N_2_ physisorption curves (**A**), XRD patterns of reduced catalysts (**B**) and TEM micrographs of reduced Co/10CeO_2_-SBA-15 (**C**) and Co/20CeO_2_-SBA-15 (**D**). Adapted from [38].

Elemental analysis confirmed that the metal contents achieved in the calcined catalysts were close to the nominal values, stating a successful incorporation of Ce and Co during synthesis. The N_2_ physisorption confirmed that the ordered mesoporous structure of SBA-15 was retained upon cerium and cobalt impregnation as in both cases the isotherms revealed a type IV isotherm with H1 hysteresis loop (see Figure 1A). However, a progressive reduction in BET area and pore volume was observed with increasing Ce content, attributed to partial pore blockage. Pore diameter determined from the maximum BJH distribution also decreased but remains within a narrow distribution due to the highly ordered structure of SBA-15, with values close to those typically reported for this material.

Reducibility was examined by H_2_-TPR. Ceria-modified catalysts exhibited a low-temperature peak associated with simultaneous Co^3+^ and Co^2+^ reduction, facilitated by ceria’s oxygen storage capacity. This behavior reflects enhanced oxygen transfer and vacancy formation, which promotes cobalt reduction. Additionally, a shift of the high-temperature peak toward higher values suggested stronger metal-support interactions in Ce-containing samples, particularly for Co/10CeO_2_-SBA-15 which also reached the highest reduction degree.

As shown in Figure 1B, the activated catalysts were analyzed by X-ray diffraction, where the absence of signals related to cobalt oxide species and the presence of Co^0^ reflection at 2θ = 44.1 and 76.4° (JCPDS-15-0806) in the XRD patterns confirmed the successful reduction in the active phase. Crystallite sizes estimated using the Scherrer equation indicated that increasing cerium content led to smaller cobalt particles beyond the detection limit (<3 nm) as no diffraction lines were observed for the sample with a 20 wt.% of Ce, suggesting improved dispersion over the support. TEM analysis shown in Figure 1C,D for Co/10CeO_2_-SBA-15 and Co/20CeO_2_-SBA-15, respectively, supported these findings, revealing the preservation of the ordered mesostructured typical for SBA-15 with the presence of dark regions corresponding to Co^0^ and CeO_2_ particles. Comparing both samples, the particle size distribution of Co/20CeO_2_-SBA-15 revealed an increase in the concentration of particles with diameters around 3 nm, consistent with the mean crystallite sizes determined by XRD.

**Figure 2:**
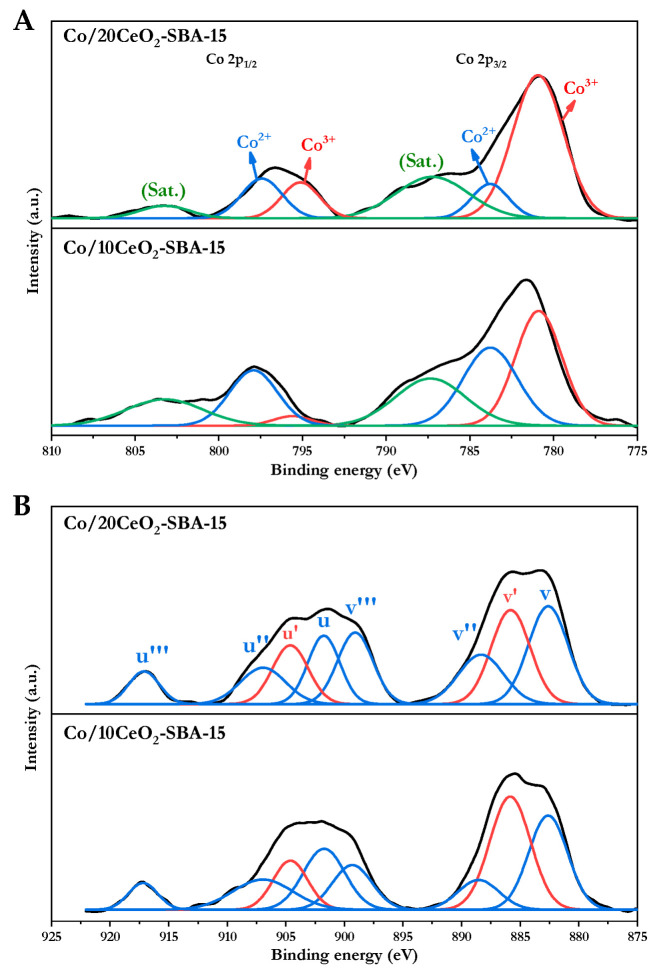
Co 2p XPS spectra (**A**) and Ce 3d XPS spectra (**B**) of Co/10CeO_2_-SBA-15 and Co/20CeO_2_-SBA-15 catalysts.

Additionally, XPS analysis was performed on the calcined catalysts to characterize in detail the surface oxidation states of cobalt and cerium oxides. The XPS Co 2p spectra shows the characteristic doublet corresponding to the Co 2p_3/2_—between 778 and 792 eV—and Co 2p_1/2_ states—between 793 and 807 eV—including the characteristic satellite peak located 5–6 eV above the main Co^2+^ peak, which is typical of cobalt in oxidized states [44], as displayed in Figure 2A. In the Co 2p spectra, the Co^2+^/Co^3+^ ratio was used as a comparative indicator of the cobalt surface redox state, which can be associated with its reducibility properties [45]. The results revealed differences between catalysts with different CeO_2_ loadings. For the Co/10CeO_2_-SBA-15 catalyst, a Co^2+^/Co^3+^ ratio of 1.2—based on the areas integrated in the spectra deconvolution—indicates that the surface cobalt was mainly in Co^2+^ state, whereas in the Co/20CeO_2_-SBA-15 catalyst, this ratio decreased to 0.3, suggesting a stabilization of more oxidized species. These findings are consistent with H_2_-TPR analysis, as the higher Co^2+^/Co^3+^ ratio correlated with the greater reduction degree of Co/10CeO_2_-SBA-15.

On the other hand, Figure 2B shows the Ce 3d spectrum, which exhibits the characteristic multiplet structure of cerium oxide formed by the spin–orbit components 3d_5/2_ (v) and 3d_3/2_ (u), appearing between 880–899 eV and 900–920 eV, respectively. The deconvolution allows the identification of six peaks associated with Ce^4+^ (v, v″, v‴, and u, u″, u‴), characteristic of CeO_2_, as well as two additional signals attributable Ce^3+^ (v′ and u′) [46,47]. The Ce^3+^/Ce_Total_ ratio was used as an indicative to measure the oxygen vacancy density on the surface. Similar Ce^3+^/Ce_Total_ ratio were obtained with both samples with a slight decrease in Co/20CeO_2_-SBA-15 (Ce^3+^/Ce_Total_ = 0.30) compared to Co/10CeO_2_-SBA-15 (Ce^3+^/Ce_Total_ = 0.30), suggesting a slight decrease in the oxygen vacancy density on the surface [47] which may result in lower oxygen mobility.

### 3.2. Oxidative Steam Reforming of Individual Model Compounds

The catalytic performance of Co/10CeO_2_-SBA-15 and Co/20CeO_2_-SBA-15 was evaluated through the oxidative steam reforming of five model compounds—acetic acid, methanol, hydroxyacetone, phenol, and furfural, selected for their representativeness in the bio-oil aqueous fraction [18]. The main reactions involved in this process are the steam reforming reactions of each model compound, according to Equations (5)–(9):

(5) $$C_{2}H_{4}O_{2}\;{+\;2H}_{2}O\;↔\;{2\mathrm{CO}}_{2}\;+\;4H_{2}$$

(6) $$CH_{3}\mathrm{OH}+H_{2}O\;↔\;{\mathrm{CO}}_{2}+3H_{2}$$

(7) $$C_{3}H_{6}O_{2}+4H_{2}O\;↔\;{3\mathrm{CO}}_{2}+7H_{2}$$

(8) $$C_{6}H_{6}O+11H_{2}O\;↔\;{6\mathrm{CO}}_{2}+{14H}_{2}$$

(9) $$C_{5}H_{4}O_{2}+8H_{2}O\;↔\;{5\mathrm{CO}}_{2}+10H_{2}$$

**Figure 3:**
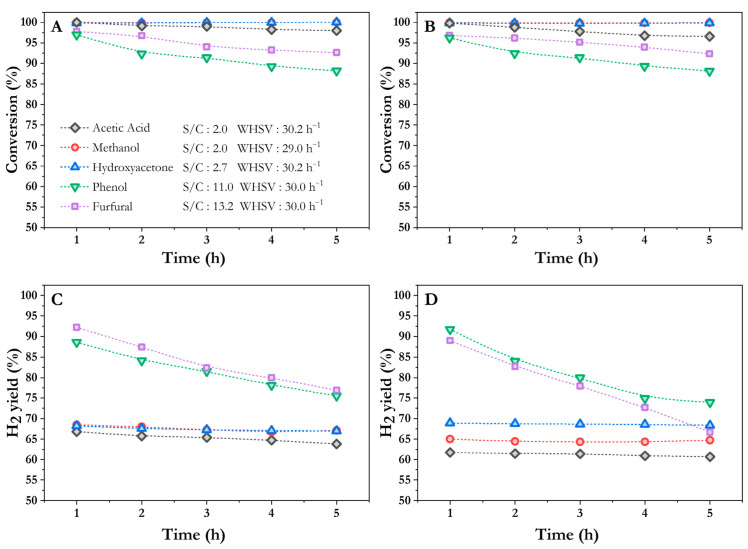
Catalytic performance of Co/10CeO_2_-SBA-15 (**A**,**C**) and Co/20CeO_2_-SBA-15 (**B**,**D**) in the oxidative steam reforming of oxygenated model compounds at 550 °C and *p* = 1 atm.

Figure 3 shows the conversion and hydrogen yield (YH_2_) over time using both catalysts. Regarding Co/10CeO_2_-SBA-15 catalyst, from Figure 3A, it is possible to observe that, in all cases, initial conversions were close to 100%, confirming the high activity of the catalysts under the operating conditions [38]. Nevertheless, after 5 h of reaction, differences emerged depending on the molecular structure of each compound. During the oxidative steam reforming of aliphatic compounds (methanol, hydroxyacetone and acetic acid), the catalyst maintained nearly complete conversion throughout the test. This behavior aligns with previous findings [38] and it is attributed to the high dispersion of cobalt particles enhanced by Ce incorporation into the SBA-15 framework. This may facilitate the cleavage of C–H and C–C bonds present in their chemical structure. Additionally, the prepared catalysts retained a high BET surface area, characteristic of well-ordered mesoporous materials, which helped to minimize diffusional limitations, thus contributing to maintaining the catalyst’s stability. In contrast, when using phenol as a model compound, a decrease of around 8% in conversion is observed after 5 h on stream. This behavior can be attributed to the inherent stability of aromatic rings and the associated difficulty in cleaving C–C and C–H bonds within these structures, due to π-electron delocalization. Moreover, aromatic compounds usually tend to form strongly adsorbed intermediates on the metal surface [48]. If these intermediates do not readily desorb, they might reduce the number of available active sites, which would explain the observed decrease in conversion. When using furfural, a decrease in conversion can also be observed because of its aromatic furan ring. In this context, Artetxe et al. [49] reported comparable behavior during the steam reforming of phenol and furfural, achieving similar activity for both model compounds, reaching lower carbon conversions for phenol, attributed to the formation of more stable intermediates during the reforming process.

This trend observed in conversion is similar to that observed for hydrogen yield (Figure 3C). Aliphatic compounds—acetic acid, methanol, and hydroxyacetone—maintained a relatively stable hydrogen yield. In contrast, compounds containing aromatic rings—phenol and furfural—exhibited a progressive decline in the hydrogen yield over time despite the higher steam-to-carbon ratios used, suggesting that the cleavage of C–H bonds and the release of hydrogen from aromatic rings is more limited than from aliphatic compounds. These constraints could drive forward the reaction toward less favorable pathways for hydrogen production [50]. When comparing aliphatic and aromatic compounds, higher hydrogen yields were obtained for the latter because higher S/C ratios were applied to overcome their lower solubility in water, thereby shifting the thermodynamic equilibrium and favoring hydrogen production.

In the case of the Co/20CeO_2_–SBA-15 catalyst, the corresponding results for each model compound, in terms of conversion and hydrogen yield with time-on-stream, are displayed in Figure 3B,D, respectively. When comparing the performance of catalysts containing 20 and 10 wt.% of Ce, minor differences were observed with the catalyst containing 10 wt.% of Ce showing slightly higher hydrogen yields which can be attributed to its slightly greater reducibility and oxygen mobility, as determined by H_2_-TPR and XPS, respectively. These findings suggest that increasing the Ce content beyond 10 wt.% does not significantly affect the catalytic activity under the tested conditions. Consequently, Co/10CeO_2_-SBA-15 was selected as the reference catalyst for this study.

In addition to hydrogen, oxidative steam reforming of oxygenated compounds also produces various carbon-containing byproducts, such as carbon monoxide, carbon dioxide, methane, and acetone. In this respect, the carbonaceous product selectivities for each model compound are shown in Table 2.

Products distribution indicates that besides the steam reforming reactions of each model compound, secondary reactions and water–gas shift reaction [16,51,52,53,54] are also taking place. In this respect thermal decomposition of acetic acid (Equations (10) and (11)), methanol (Equation (12)) and hydroxyacetone (Equation (13)) must be considered which produce CO and CH_4_. The CO produced can either be converted into hydrogen via the water–gas shift reaction (Equation (14)) or consume hydrogen to form methane through the methanation reaction (Equation (15)). In turn, methane once formed, can undergo steam reforming to yield CO_2_ and H_2_ according to Equation (16). In addition to these reactions, considering that oxygen is fed into the system, it can react with reactants or reaction intermediates through oxidation reactions.

(10) $$C_{2}H_{4}O_{2}\;↔\;2\mathrm{CO}+2H_{2}$$

(11) $$C_{2}H_{4}O_{2}\;↔\;CO_{2}+CH_{4}$$

(12) $$CH_{3}\mathrm{OH}\;↔\;\mathrm{CO}+2H_{2}$$

(13) $$C_{3}H_{6}O_{2}\;↔\;{\mathrm{CH}}_{4}+2\mathrm{CO}+H_{2}$$

(14) $$\mathrm{CO}+H_{2}O\;↔\;{\mathrm{CO}}_{2}+H_{2}$$

(15) $$\mathrm{CO}+3H_{2}\;↔\;{\mathrm{CH}}_{4}+H_{2}O$$

(16) $${\mathrm{CH}}_{4}+{2H}_{2}O\;↔\;CO_{2}+4H_{2}$$

For these compounds, the tendency for product distribution was characterized by clear predominance of CO_2_ (62–75%), followed by CO (23–36%), with low CH_4_ formation (2–5%). It is noteworthy that both acetic acid and hydroxyacetone tend to form more CO_2_ than methanol. Both contain carbonyl groups, in which the carbon atom is already in a highly oxidized state. This could make them more prone to complete oxidation to CO_2_ via decarboxylation. In line with this observation, Bkangmo Kontchouo et al. [55] investigated the steam reforming of several oxygenated compounds—ethanol, acetaldehyde, acetone and acetic acid—and reported higher CO_2_ yields for those containing carbonyl groups compared to ethanol. Additionally, both compounds produce small amounts of acetone, suggesting that condensation or molecular rearrangement reactions are also taking place according to Equations (17) and (18). This effect is more pronounced with hydroxyacetone, likely due to the presence of both carbonyl and hydroxyl functional groups, which enable acetone formation via dehydrogenation of the hydroxyl group.

(17) $$2C_{2}H_{4}O_{2}\;↔\;C_{3}H_{6}O+CO_{2}+H_{2}O$$

(18) $$C_{3}H_{6}O_{2}\;+H_{2}\;↔\;C_{3}H_{6}O\;+H_{2}O$$

Regarding CH_4_ formation, higher values were achieved when reforming acetic acid and hydroxyacetone compared to methanol, which could be attributed to the fact that methane formation is limited to methanation reaction (Equation (15)). In contrast, methane formation during acetic acid OSR may result not only from methanation reaction but also from the thermal decomposition reaction (Equation (11)). For hydroxyacetone, thermal decomposition (Equation (13)) further contributes to methane generation. The higher methane formation observed for hydroxyacetone is consistent with the findings reported by Palmeri et al. [56] who observe that compounds containing a greater number of carbon atoms bounded to carbonyl oxygen tend to exhibit higher methane selectivity.

In the case of phenol and furfural, the product distribution consisted exclusively of CO and CO_2_, with no detectable traces of CH_4_. This absence of CH_4_ can be attributed to the high steam/carbon ratios used (S/C = 11 for phenol and 13.2 for furfural), which were necessary to ensure their solubility in water. Under high S/C ratios, the concentration of steam in the feed stream increases, thus disrupting the thermodynamic equilibrium of methanation reaction (Equation (15)), making them unfavorable. This shifts the reaction pathway towards H_2_, CO and CO_2_ products [57], while effectively inhibiting the formation of CH_4_ as reported elsewhere [16]. On the other hand, furfural exhibited higher CO_2_ selectivity compared to phenol, which could be attributed to the presence of the formyl group in its structure. This functional group may be more prone to oxidation under steam reforming conditions, possibly due to its electronic characteristics (partial positive charge on the carbonyl carbon) and lower stability compared to the hydroxyl group in phenol [58].

**Figure 4:**
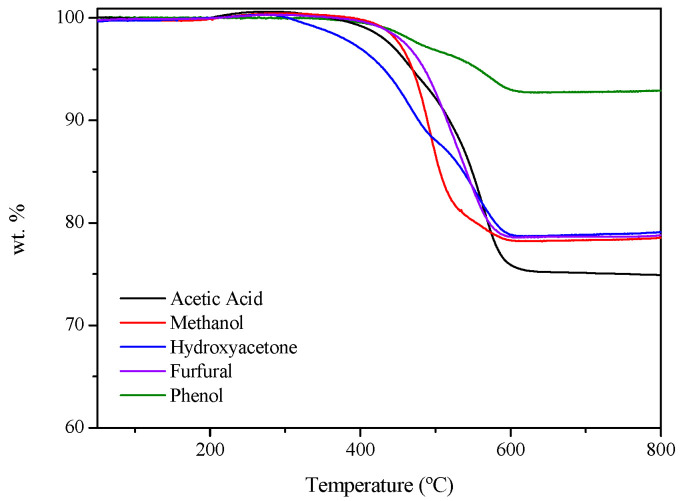
TGA profiles of the spent catalysts during the OSR of model compounds.

In addition to conversion and product distribution, coke formation must also be considered because it arises from undesirable reaction pathways, and it can ultimately lead to catalyst deactivation. In this respect, Figure 4 shows the curve obtained in TGA and Table 3 summarizes the coke characterization of the spent catalyst, including coke formation rate, coke yield and the temperature range of weight loss for all the model compounds tested. In all cases, the weight loss falls within the range of 330–650 °C with maximum rates between 470 and 565 °C, which evidences the formation of carbon nanofilaments, as amorphous carbon typically oxidizes at lower temperatures [24]. For acetic acid, methanol, and furfural, the decomposition occurs with a single dominant change in slope, suggesting the formation of a relatively homogeneous coke, likely composed of filamentous carbon structures that oxidize within a narrow temperature range. In contrast, hydroxyacetone and phenol display a more gradual weight loss with two different stages, which suggest the presence of at least two carbon species with different ordering degrees.

It is noteworthy that phenol and furfural exhibited higher coke yields (Y_coke_)—defined as the ratio between the moles of coke deposited per unit of time during the whole test and the molar flow rate of C fed for each compound—compared to the aliphatic compounds. This increased tendency is attributed to their higher stability and the presence of aromatic structures, which promote polymerization reactions that ultimately lead to coke formation. This assumption is consistent with the findings of Remón et al. [59], who investigated the influence of bio-oil composition during steam reforming and identified furfural and phenolic compounds as the main contributors to coke deposition on the catalyst surface.

### 3.3. Oxidative Steam Reforming of Bio-Oil Model Compound Mixtures

#### 3.3.1. OSR of Aqueous Mixture of Three Model Compounds

**Figure 5:**
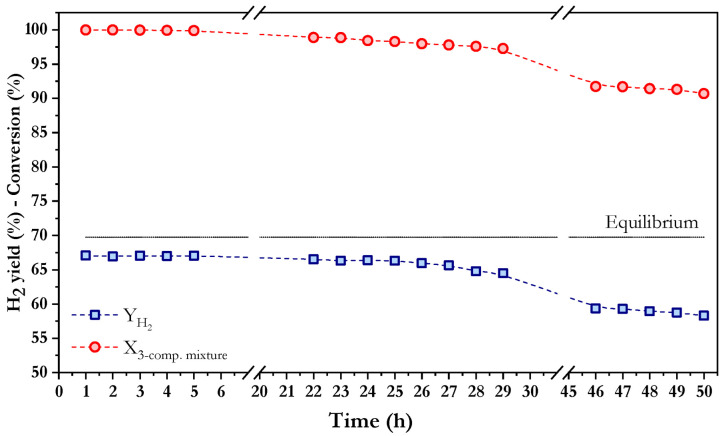
Evolution of total conversion and hydrogen yield with time in oxidative steam reforming of a mixture of 3 model compounds using Co/10CeO_2_-SBA-15 at 550 °C, S/C = 2.29, WHSV = 30.11 h^−1^, and *p* = 1 atm.

Trying to approach bio-oil aqueous phase complex composition, mixtures of three and five model compounds were prepared and tested in oxidative steam reforming using Co/10CeO_2_-SBA-15. A mixture of methanol (2.10 wt.%), hydroxyacetone (15.50 wt.%), and acetic acid (22.78 wt.%) was prepared based on the results reported by Bergem et al. [17]. Thereby, Figure 5 shows the catalytic performance of Co/10CeO_2_-SBA-15 during a stability test using this three-component mixture evaluated in terms of conversion and hydrogen yield. The results indicate excellent catalytic performance during the first 25 h on stream, with nearly complete conversion and hydrogen yields around 67%, which is close to the value predicted by the thermodynamic equilibrium under the tested conditions. From this point onward, a slight deactivation can be observed, as evidenced by the progressive decrease in conversion and hydrogen yield. Nevertheless, the results obtained after 50 h—approximately 90% total conversion with a hydrogen yield of 58%—remain satisfactory, confirming that the catalyst retains good activity for the reforming of these aliphatic compound mixtures under long-term reaction conditions. This suggests that, despite the interactions between these compounds could lead to the formation of intermediate species, they have a minimal impact on overall catalytic performance.

**Figure 6:**
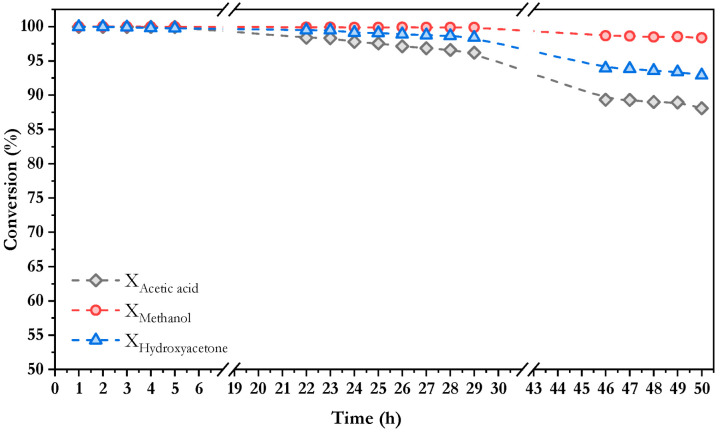
Conversion values along time on stream for each model compound in the oxidative steam reforming of a mixture of 3 model compounds using Co/10CeO_2_-SBA-15 catalyst at 550 °C, S/C = 2.29, WHSV = 30.11 h^−1^, and *p* = 1 atm.

To further investigate the catalytic results, Figure 6 displays the partial conversions of each compound along time on stream. As shown, methanol exhibits the highest values, followed by hydroxyacetone, and finally acetic acid, which reaches a conversion of nearly 90% after 50 h on stream. These differences in conversion after 30 h on stream may be primarily attributed to the effective diffusivity of each compound within the catalyst porous structure, calculated as a function of diffusivity, porosity and tortuosity according to Equation (19) [60].

(19) $$D_{e}=\frac{D\cdot \varepsilon }{\tau }$$

where D represents the diffusivity in m^2^/s, ε is the catalyst porosity and τ refers to tortuosity. In porous catalysts, this overall diffusion rate is influenced by two mechanisms: molecular diffusion (D_A_) and Knudsen diffusion (D_K_). Molecular diffusion describes the transport of species through collisions in the gas phase, while Knudsen diffusion becomes significant when the pore size is comparable to or smaller than the mean free path of the molecules [61]. To accurately predict mass transport inside the pores, both contributions must be considered according to the Bosanquet equation (Equation (20)).

(20) $$D=\frac{1}{\left(\frac{1}{D_{k}}+\frac{1}{D_{A}}\right)}$$

D_K_ for each model compound was estimated according to Equation (21):

(21) $$D_{K}=\frac{2}{3}\cdot r\cdot \sqrt{\frac{8\mathrm{RT}}{\pi \cdot M}}$$

where r is related to the radius of catalyst pores (4.45 × 10^−9^ m), R is the universal gas (8.314 J·mol^−1^·K^−1^), T is the reaction temperature (K), and M is the molecular weight of each component (kg/mol). Based on this method, the estimated coefficients are 1.48 × 10^−6^ m^2^/s for methanol, 1.08 × 10^−6^ m^2^/s for acetic acid, and 9.70 × 10^−7^ m^2^/s for hydroxyacetone.

The molecular diffusion coefficient of acetic acid, methanol and hydroxyacetone in the gas phase was estimated using the Chapman–Enskog theory (Equation (22)), which provides a rigorous framework for predicting transport properties in gases [61].

(22) $$D_{A}=\frac{3}{16}\cdot \frac{{(k}_{B}\cdot {T)}^{3/2}}{p\cdot \sigma^{2}\cdot \sqrt{\pi \cdot m}}$$

where k_B_ is the Boltzmann constant (1.38 × 10^−23^ J/K), T is the temperature (K), *p* is the pressure (Pa), σ is the molecular diameter of each compound (m), and m the mass of a single molecule (M/N_A_) in kg. The calculated molecular diffusion coefficients are approximately 4.2 × 10^−5^ m^2^/s for methanol, 1.6 × 10^−5^ m^2^/s for acetic acid, and 1.7 × 10^−5^ m^2^/s for hydroxyacetone. The calculated value was then combined with Knudsen diffusion using the Bosanquet equation (Equation (20)) to determine the effective diffusivity (Equation (19)) within the porous catalyst. The porosity of the Co/10CeO_2_-SBA-15 catalyst was determined using Equation (23), based on the experimentally measured bulk and tap densities of 0.0915 g/cm^3^ and 0.170 g/cm^3^, respectively. The calculated porosity was 0.462.

(23) $$\varepsilon =\;1-\;\frac{\rho_{\mathrm{bulk}}}{\rho_{\mathrm{tap}}}$$

Tortuosity was estimated from the catalyst porosity using the Bruggeman equation (Equation (24)), a widely employed method for correlating tortuosity with the porosity of the materials [62].

(24) $$\tau =\gamma \cdot \varepsilon^{\;(1-\alpha )}$$

In this equation, the parameters γ and α are constants that reflect the influence of the material’s morphology, composition, and particle-size distribution. For porous materials, typical values for these constants are γ = 1 and α = 1.5 [62,63], resulting in a tortuosity of 1.47. Once all the required parameters were determined, the effective diffusivity was calculated using the previously mentioned equation (Equation (19)), resulting in values of 4.49 × 10^−7^ m^2^/s for methanol, 3.18 × 10^−7^ m^2^/s for acetic acid, and 2.88 × 10^−7^ m^2^/s for hydroxyacetone. Considering that methanol has the highest effective diffusivity, it is reasonable to affirm that it would access the active sites of the catalyst and undergo reforming more easily than the others, which explains the highest conversion achieved throughout the test. Additionally, this behavior may be due to the absence of C–C bonds in methanol, which eliminates the need for bond cleavage and simplifies the reforming process. Based on this same assumption, acetic acid would be expected to achieve a higher conversion than hydroxyacetone, given the higher effective diffusivity. Nevertheless, the experimental results do not support this theory. This discrepancy can be ascribed to the molecular stability of both compounds. Hydroxyacetone, despite its lower effective diffusivity, is less stable than acetic acid due to the presence of a central carbonyl group next to a hydroxyl group, making it more reactive and prone to decomposition, which could explain its higher conversion than acetic acid.

**Figure 7:**
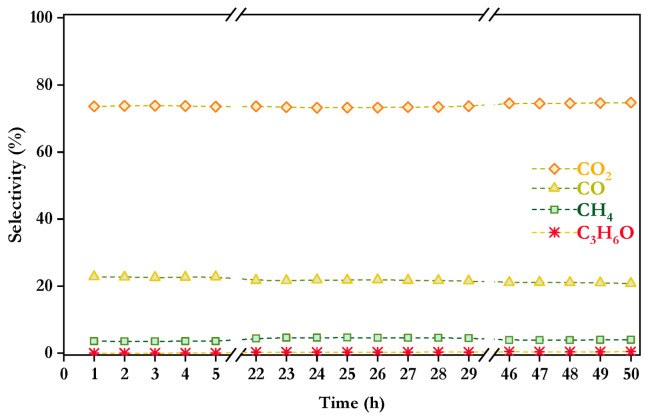
Selectivity to carbonaceous products over Co/10CeO_2_-SBA-15 after 50 h time-on-stream in the mixture of 3 model compounds at 550 °C, S/C = 2.29, WHSV = 30.11 h^−1^, and *p* = 1 atm.

The selectivities to carbon co-products for 50 h on stream are displayed in Figure 7. As can be observed, only minor variations were detected for all carbon-containing co-products throughout the entire test, with selectivity values at 50 h of 3.96% for methane, 20.82% for CO, 74.73% for CO_2_, and 0.49% for acetone. The oxidative steam reforming of a 3-component model mixture showed differences in carbon co-product distribution compared to individual components (see Table 2), with lower CO selectivity and higher CO_2_ selectivity, which may suggest that the contribution of the water–gas shift reaction (Equation (14)) is favored, converting CO and H_2_O into CO_2_ and H_2_. In addition, the coexistence of different oxygenated compounds could potentially lead to molecular interactions that alter the reaction network. Such interactions might result in the formation of intermediate hydrocarbons, which are further oxidized by OH radicals, producing H_2_ and CO [64]. From another point of view, the formation of oxygenated intermediates that could act as internal oxidants would be possible, thereby enabling the transformation of CO into CO_2_. On the other hand, the selectivity to methane is intermediate between the values obtained for each model compound (ranging from 1.83% achieved with methanol to 4.94% with hydroxyacetone), which may indicate that methanogenic pathways were neither significantly promoted nor inhibited under the tested conditions. Therefore, the obtained results did not demonstrate a clear synergistic effect related to the potential interactions between methanol, acetic acid and hydroxyacetone with respect to methane formation. Finally, an increase in acetone selectivity was observed, which may suggest the occurrence of condensation or dehydration reactions between intermediates, potentially involving carbonyl and hydroxyl groups from hydroxyacetone and acetic acid as explained above.

When reforming the ternary mixture, a coke yield of 0.253% was obtained and the maximum rate in the TG curve of the used catalyst is around 550 °C. These values are consistent with the trends observed for the individual model compounds, as both the coke yield and maximum rate are comparable to those reported in Table 3, suggesting the predominance of filamentous carbon structures.

#### 3.3.2. OSR of Five Models Compound Aqueous Mixture

**Figure 8:**
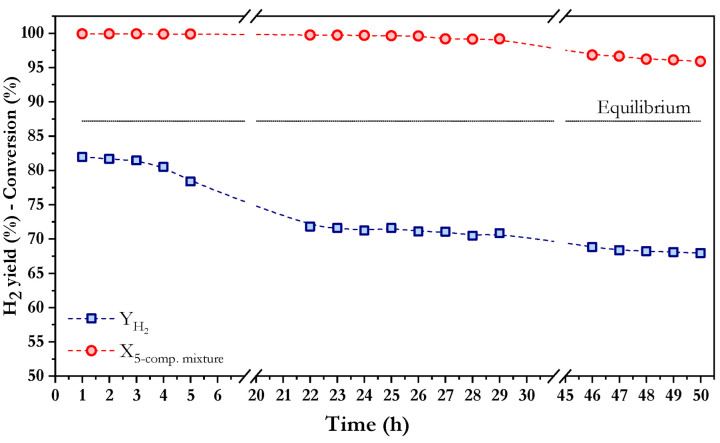
Evolution of total conversion and hydrogen yield with time in oxidative steam reforming of a mixture of 5 model compounds using Co/10CeO_2_-SBA-15 at 550 °C, S/C = 6, WHSV = 30 h^−1^, and *p* = 1 atm.

Although a ternary mixture of representative aliphatic compounds from the bio-oil aqueous phase provides more information than using a single model compound, it remains a simplified approach, given that real fractions are considerably more complex. They typically contain a wide variety of functional groups, including aromatic and heterocyclic compounds. To better reflect its complexity, a mixture of five model compounds is proposed, incorporating methanol, acetic acid, hydroxyacetone, phenol and furfural. This expanded formulation enables a more realistic evaluation of catalyst performance under oxidative steam reforming conditions. Based on the composition described by Fermoso et al. [18], the mixture with a S/C ratio of 6 contains 7.91 wt.% acetic acid, 1.25 wt.% methanol, 7.19 wt.% hydroxyacetone, 0.84 wt.% phenol, 1.92 wt.% furfural, and 80.88% water. The higher S/C ratio used compared to the 3-compound mixture, apart from being necessary given the low solubility of phenol and furfural in water, resembles the high-water concentration present in the real bio-oil aqueous fractions [18]. The results for conversion and hydrogen yield over time are displayed in Figure 8. From this figure, it is possible to see how the Co/10CeO_2_SBA-15 catalyst maintained nearly complete conversion throughout the test, with values above 99% during the first 30 h of reaction, followed by a slight decrease of approximately 4% after 50 h on stream. Regarding the hydrogen yields, an initial value of 82% was achieved within the first hours, followed by a progressive decrease that stabilized around 70% from 20 to 30 h, with a slight decrease when the time on stream is higher than 45 h. This trend reveals good Co/10CeO_2_SBA-15 catalyst stability.

**Figure 9:**
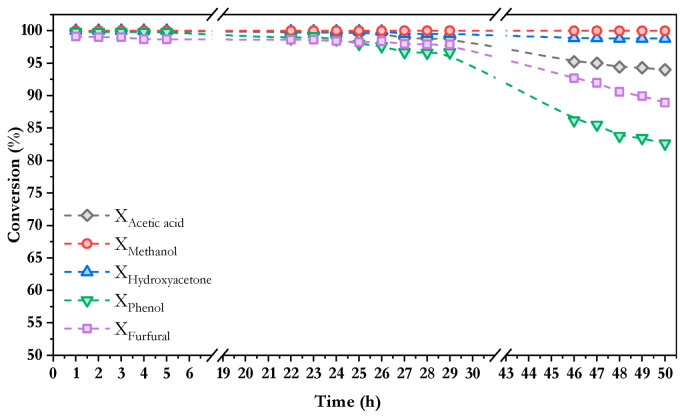
Evolution of conversion for each model compound in the mixture of 5 model compounds with time in oxidative steam reforming of a using Co/10CeO_2_-SBA-15 at 550 °C, S/C = 6, WHSV = 30 h^−1^, and *p* = 1 atm.

In the same way as for the three-model compound mixture, the partial conversion for each compound is shown in Figure 9. The aliphatic compounds—methanol, hydroxyacetone, and acetic acid—followed the same trend observed previously with conversions above 94% in all cases, suggesting that the presence of aromatic compounds in the feed mixture did not significantly affect the reactivity of these species under the tested conditions. In contrast, during the final hours of the reaction, the aromatic compounds exhibited considerably lower conversion rates than methanol, acetic acid and hydroxyacetone, reaching values of approximately 89% for furfural and 83% for phenol after 50 h on stream. As discussed above, the lower conversion for furfural and phenol is associated with the intrinsic chemical stability of aromatic rings, which makes the cleavage of C–C and C–H bonds more difficult compared to the other compounds. For the same reasons explained above, furfural exhibited higher conversion than phenol in the same way as was observed in Figure 3.

**Figure 10:**
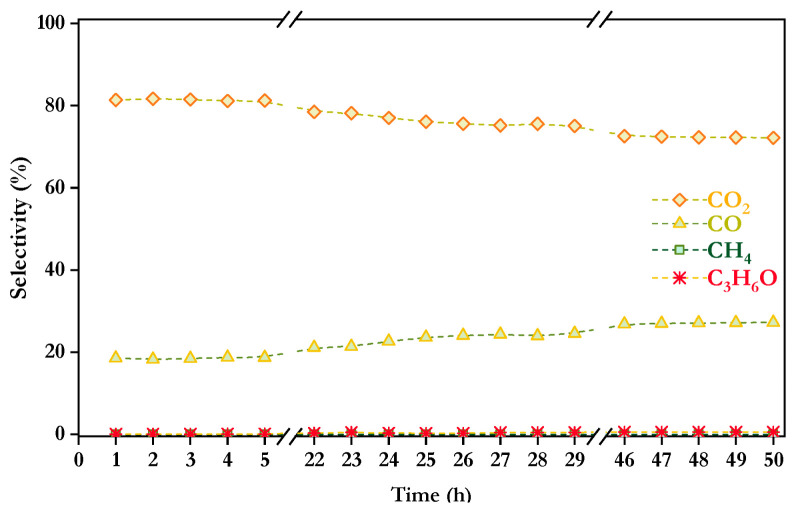
Selectivity to carbonaceous products over Co/10CeO_2_-SBA-15 after 50 h time-on-stream in the mixture of 5 model compounds at 550 °C, S/C = 6, WHSV = 30 h^−1^, and *p* = 1 atm.

Carbon co-products selectivities are shown in Figure 10. Slight variations for CO and CO_2_ are observed over time, with selectivity values of 27.26% for CO, 72.14% for CO_2_ and 0.59% for acetone after 50 h. It is noteworthy that, in contrast with the mixture of 3 model compounds, methane formation was absent. As previously discussed, increasing the S/C ratio, in this case to favor phenol and furfural solubility in water, raised the steam concentration in the feed, which shifted the equilibrium of methanation reaction (Equation (15)) towards the reactants, favoring the formation of H_2_, CO and CO_2_ [16]. This effect also accounted for the higher formation rate of CO—compared to the product distribution with the mixture of 3 model compounds. However, acetone selectivity slightly increased compared to the result obtained with the mixture of 3 model compounds. Although the increase in acetone production was not as pronounced as in the previous case, an increase in the formation of reaction intermediates may be expected with the inclusion of furfural and phenol in the feed mixture. This can lead to increased interactions between compounds, potentially promoting condensation reactions that contribute to acetone formation.

In the case of the five-compound mixture, a coke yield of 0.354 with two maximum rates within the temperature range of 450–550 °C were obtained. Compared to the ternary mixture, these values indicate a slightly higher tendency for coke formation. This increase is associated with the presence of phenol and furfural, as both compounds typically generate more coke than aliphatic species, as previously discussed. The maximum temperature rates achieved again evidence the formation of filamentous carbon structures with varying ordering degrees.

Previous studies on oxidative steam reforming of acetic acid have primarily focused on Ni-based catalysts using a single model compound. However, given the limited amount of oxygen supplied, a comparison with Co-based catalysts under steam reforming conditions can be considered. Although such comparison is challenging due to differences in operating conditions, the results presented in Table 4, underline the potential of Co/10CeO_2_-SBA-15 as an effective catalyst for the oxidative steam reforming of bio-oil aqueous fractions, demonstrating high activity and hydrogen selectivity.

## 4. Conclusions

This work demonstrates the excellent catalytic performance of Co/CeO_2_-SBA-15 catalysts to obtain renewable hydrogen through the oxidative steam reforming of model compound mixtures simulated bio-oil aqueous fractions. The OSR of each model compound reflects how aliphatic model compounds like methanol, acetic acid and hydroxyacetone achieved near-complete conversion and stable hydrogen yields throughout the reaction. In contrast, aromatic compounds (furfural and phenol) showed slightly lower conversion and hydrogen yields, attributed to their structural stability and tendency to form strongly adsorbed intermediates, resulting in higher coke yields. No significant differences were found when increasing catalysts ceria loading from 10 to 20%, so Co/10CeO_2_-SBA-15 was subsequently used in the OSR of model compound mixtures. The ternary mixture composed of methanol, hydroxyacetone, and acetic acid maintained high conversion and hydrogen yield over 50 h on stream. When furfural and phenol were added to the ternary mixture, their presence did not affect the reactivity of aliphatic compounds. However, their individual conversions were notably lower, although they remained above 80% after 50 h on stream in both cases. Therefore, these findings confirm that Co/10CeO_2_-SBA-15 is a viable catalyst for hydrogen production during the oxidative steam reforming of complex bio-oil aqueous fractions.

## Figures and Tables

**Table 1:** Physicochemical characterization of the prepared Co/CeO2-SBA-15 catalysts.

Sample	Ce ^a^	Co ^a^	S_BET_	Vp ^b^	Dp ^c^	DCo^0 d^	Reducibility
	(wt. %)	(wt. %)	(m^2^/g)	(cm^3^/g)	(nm)	(nm)	(%)
Co/10CeO_2_-SBA-15	12.3	7.4	344	0.77	8.9	4.2	~100
Co/20CeO_2_-SBA-15	23.3	7.1	303	0.62	8.2	n.d. ^e^	97

^a^ Measured by ICP-OES in calcined catalysts. ^b^ Calculated at P/P_0_ = 0.95. ^c^ BJH pore size distribution maximum. ^d^ Determined by (111) reflecting plane of the Co^0^ pattern in XRD of reduced samples. ^e^ Not detectable. Equipment detection range (>3 nm).

**Table 2:** Selectivity to carbonaceous products over Co/10CeO_2_-SBA-15 after 5 h time-on-stream at 550 °C and *p* = 1 atm.

Compound	CH_4_ (%mol)	CO (%mol)	CO_2_ (%mol)	Acetone (%mol)
Acetic Acid	2.51	22.69	74.66	0.14
Methanol	1.83	36.04	62.13	0.00
Hydroxyacetone	4.94	23.48	71.38	0.19
Phenol	0.00	27.67	72.33	0.00
Furfural	0.00	19.58	80.42	0.00

**Table 3:** Coke characterization of the spent Co/10CeO_2_-SBA-15 catalyst after oxidative steam reforming of each model compound. T^a^ = 550 °C, *p* = 1 atm, t = 5 h.

Compound	Coke Deposition (mg_coke_/g_cat_·h)	Y_coke_ (%mol)	T_range, TG_ (°C)
Acetic Acid	7.3	0.27	330–650
Methanol	5.1	0.21	340–630
Hydroxyacetone	5.5	0.22	300–620
Phenol	3.6	0.43	330–640
Furfural	5.9	0.84	360–620

**Table 4:** Catalytic performance of several catalysts in bio-oil aqueous fraction steam reforming.

Catalyst	Reaction Type/Feedstock	Operating Conditions	H_2_ Yield/Conversion	Reference
Co/SBA-15	Steam reforming/Simulated bio-oil aqueous phase: phenol, hydroxyacetone, and acetic acid	T = 600 °C; S/C = 1.1; WHSV = 30.2 h^−1^; TOS = 50 h	H_2_ yield = 57%/Conversion = 80%	[24]
Ni-Co/SBA-15	Steam reforming/Simulated bio-oil aqueous phase: acetic acid, hydroxyacetone, phenol, and furfural	T = 600 °C; S/C = 0.95; W_cat_ = 300 mg; TOS = 50 h	H_2_ yield = 56%/Conversion > 95%	[16]
CoAl_2_O_4_	Steam reforming/Simulated bio-oil aqueous phase: ethanol, acetic acid, acetone, and phenol	T = 700 °C; S/C = 3; WHSV = 10.6 h^−1^; TOS = 4 h	H_2_ yield = 30%/Conversion = 60%	[65]
Co/Sepiolite	Steam reforming/Simulated bio-oil aqueous phase: ethanol, acetic acid, acetone, and phenol	T = 700 °C; S/C = 3; WHSV = 10.6 h^−1^; TOS = 50 h	H_2_ yield = 63%/Conversion = 76%	[65]
Co-Ni/Biochar	Steam reforming/Slow-pyrolysis bio-oil	T = 700 °C; S/C = 3.87; LHSV = 1.47 h^−1^; TOS = 13 h	H_2_ selectivity = 55%/Conversion = 65%	[66]
Co/10CeO_2_-SBA-15	Oxidative steam reforming/Simulated bio-oil aqueous phase: acetic acid, methanol, hydroxyacetone, phenol, and furfural	T = 550 °C; S/C = 6; O_2_/C = 0.0375; WHSV = 30.2 h^−1^; TOS = 50 h	H_2_ yield = 68%/Conversion = 96%	This work

## Data Availability

Open access data is available at https://doi.org/10.21950/LPVFCN.
